# Effect of enhanced external counterpulsation treatment on renal function in cardiac patients

**DOI:** 10.1186/1471-2369-14-193

**Published:** 2013-09-11

**Authors:** Prajej Ruangkanchanasetr, Nithi Mahanonda, Ongkarn Raungratanaamporn, Piyanuj Ruckpanich, Chagriya Kitiyakara, Amnart Chaiprasert, Surawat Adirekkiat, Dollapas Punpanich, Somlak Vanavanan, Anchalee Chittamma, Thanom Supaporn

**Affiliations:** 1 Division of Nephrology, Department of Medicine, Phramongkutklao Hospital, 315 Rajavithi Road, Rajathevi, Bangkok 10400, Thailand; 2 Perfect Heart Institute, Piyavate Hospital, Bangkok, Thailand; 3 Division of Nephrology, Department of Medicine, Ramathibodi Hospital, Bangkok, Thailand; 4 Division of Nephrology, Department of Medicine, Piyavate Hospital, Bangkok, Thailand; 5 Division of Clinical Chemistry, Department of Pathology, Ramathibodi Hospital, Bangkok, Thailand

**Keywords:** Angina, Chronic kidney disease, Cystatin C, Glomerular filtration rate, Heart failure

## Abstract

**Background:**

Enhanced external counterpulsation (EECP) enhances coronary perfusion and reduces left ventricular afterload. However, the role of EECP on renal function in cardiac patients is unknown. Our aim was to assess renal function determined by serum cystatin C in cardiac patients before and after EECP treatment.

**Methods:**

A prospective observational longitudinal study was conducted in order to evaluate renal function using serum cystatin C (Cys C) and estimated glomerular filtration rate (GFR) after 35 sessions of EECP treatment in 30 patients with chronic stable angina and/or heart failure. The median (IQR) time for follow-up period after starting EECP treatment was 16 (10–24) months.

**Results:**

Cys C significantly declined from 1.00 (0.78-1.31) to 0.94 (0.77-1.27) mg/L (p < 0.001) and estimated GFR increased from 70.47 (43.88-89.41) to 76.27 (49.02-91.46) mL/min/1.73 m^2^ (p = 0.006) after EECP treatment. Subgroup analysis showed that patients with baseline GFR <60 mL/min/1.73 m^2^ or NT-proBNP >125 pg/mL had a significant decrease in Cys C when compared to other groups (p < 0.01).

**Conclusions:**

The study demonstrated that EECP could improve long-term renal function in cardiac patients especially in cases with declined renal function or with high NT-proBNP.

**Trial registration:**

The study was registered in the clinical trial as International Standard Randomized Controlled Trial Number ISRCTN11560035.

## Background

A close relationship between the function of the heart and the kidneys has long been recognized. The bidirectional nature of this interaction forms an important concept in the classification of the cardiorenal syndrome. A decrease in cardiac function could adversely impact renal function [1]. Impaired renal function is independently associated with increased risk for cardiovascular diseases, hospitalization for congestive heart failure (CHF) and cardiovascular death [2,3]. Therefore, therapies that improve cardiac function might have potentially beneficial effects on renal perfusion and glomerular filtration.

EECP is a nonpharmacologic therapy for outpatients with angina pectoris and CHF [4,5]. EECP is based on the principle of diastolic augmentation to increase coronary flow while simultaneously decreasing systolic afterload. During diastole, cuffs inflate sequentially from the calves proximally to raise diastolic aortic pressure and theoretically increase coronary perfusion pressure. At the same time, increased venous return would result in increased cardiac output by Starling mechanism. Then the cuffs are rapidly decompressed at the onset of systole creating negative pressure that decreases cardiac afterload [6,7].

EECP is a low risk procedure, approved by US Food and Drug Administration for refractory angina and CHF [5,8,9]. Nevertheless, the impact of EECP treatment on renal function in cardiac patients has not been well documented. The objective of this study was to assess the effects of EECP treatment on renal function in patients with chronic stable angina and/or CHF.

## Methods

### Study design

This study was a prospective observational longitudinal study. Outpatients at cardiology clinic of Piyavate hospital were included during January 2007 and November 2009. Inclusion criteria were (1) age 18 years or more (2) having chronic stable angina and/or CHF. Exclusion criteria were (1) having unstable angina or acute myocardial infarction or decompensated CHF in the preceding one month (2) undergoing coronary angiography or coronary artery bypass grafting in the preceding one month (3) blood pressure >180/110 mmHg (4) severe symptomatic peripheral vascular disease and (5) GFR <15 mL/min/1.73 m^2^. This study was approved by the Institutional Review Board of Piyavate hospital and written informed consent was obtained from each patient. The study was registered in the clinical trial as International Standard Randomized Controlled Trial Number ISRCTN11560035.

After recruitment, all patients underwent a standard round of 35 sessions of 1-hr daily EECP treatment using EECP machine (Vasomedical, Westbury, New York, USA) over a period of 7–8 weeks. Demographics, baseline characteristics, clinical presentations and laboratory findings were collected in the case record forms. Prior to EECP treatment, fasting blood samples were collected and then also immediately collected at the end of 35^th^ session as well as every 2–3 month interval.

The samples were centrifuged at 5000 rpm for 5 minutes at 4°C and the supernatant was separated and stored at −30°C until analysis. Renal function was evaluated by changes in Cys C and estimated GFR using an equation that combined both serum creatinine and cystatin C with age, sex and race [10]. This equation has been shown to have improved accuracy compared to the estimated GFR equations that used either one of these markers alone [10].

Cr was measured based on isotope-dilution mass spectrometry standardized enzymatic method using the SRM 967 as control. The mean concentrations with the acceptable ranges of 2 SRM levels were 65.4 (66.5 ± 1.9) and 343.9 (346.2 ± 7.3) μmol/L, respectively. Cys C was measured by a particle-enhanced immunonephelometric assay using a BN Prospec nephelometer (Siemens Healthcare Diagnostics, Germany). The assay range is 0.23 mg/L to 8.00 mg/L. Within-run precision was assessed at the low (0.92 mg/L) and high (1.97 mg/L) concentrations which yielded coefficients of variation (CoV) of 1.92% and 1.08% and day-to-day CoV of 2.03% and 1.28%, respectively. NT-proBNP was also evaluated before and after 35 sessions of EECP treatment. NT-proBNP was determined by a sandwich immunoassay using Elecsys proBNP 2010 (Roche Diagnostics, Basel, Switzerland). The analytical range extends from 5–35,000 pg/mL.

### Sample size calculation

The sample size was calculated based on the report of Werner *et al.*[11] showing that the pre-EECP GFR and post-EECP GFR of 12 patients in the control group were 68 ± 16 and 84 ± 22 mL/min/1.73 m^2^. An estimated number of 26 patients would give 90% power for a statistically significant difference of the GFR outcome between pre and post EECP. To account for a 10% dropout rate, about 30 patients were enrolled.

### Statistical analysis

All statistical analysis was performed using STATA version 9.2 (StataCorp LP, Texas, USA). Continuous data were summarized as mean (SD) or median (IQR). For normally distributed data, paired T- test was used and Wilcoxon signed ranks test for non-normally distributed data. Categorical data were expressed as frequencies and percentages. All p values were two-sided. A p-value of < 0.05 was considered statistically significant.

## Results

A total of 30 patients with chronic angina (23/30, 76.7%) and/or CHF (7/30, 23.3%) were recruited to the prospective observational longitudinal study during January 2007 and November 2009. The median (IQR) follow up time was 16 (10–24) months after starting EECP treatment. Baseline characteristics, clinical parameters and laboratory findings are shown in Table 1 and Table 2. Majority of patients were male (76.7%, 23/30). The study patients had mean (SD) age of 69.1 (13.8) years and body mass index of 26.1 (4.9) kg/m^2^. Median (IQR) systolic blood pressure [(122, 113–133) mmHg], diastolic blood pressure [(75, 70–77) mmHg] and heart rate [(70, 61–75) beats/min] were within normal limits.

**Table 1:** Demographic and baseline characteristics of study patients (n = 30)

**Characteristics**	
Age (yr), mean (SD)	69.1 (13.8)
Male	23 (76.7%)
Body mass index (kg/m^2^) , mean (SD)	26.1 (4.9)
*Clinical history*	
Coronary artery disease	29 (96.7%)
Hypertension	27 (90.0%)
Diabetes	23 (76.7%)
Stroke	8 (26.7%)
Angina pectoris	23 (76.7%)
Heart failure	7 (23.3%)
Peripheral artery disease	4 (13.3%)
Smoking status	10 (33.3%)
Past smoker	7 (70.0%)
Current smoker	3 (30.0%)
*Previous procedures*	
Coronary angiography	27 (90.0%)
Percutaneous coronary intervention	12 (40.0%)
Coronary artery bypass grafting	6 (20.0%)
*Medication*	
Statin	28 (93.3%)
Aspirin or Clopidogrel	27 (90.0%)
ACE inhibitor or ARB	23 (76.7%)
Beta-blocker	19 (63.3%)
Nitrate	17 (56.7%)
Calcium-channel blocker (Diltiazem or Verapamil)	10 (33.3%)
*Laboratory findings, mean (SD)*	
Hemoglobin (g/dL)	12.9 (1.8)
Albumin (g/dL)	4.2 (0.4)
Cholesterol (mg/dL)	146.0 (27.0)
HDL cholesterol (mg/dL)	49.5 (12.2)
LDL cholesterol (mg/dL)	77.8 (19.6)
Triglyceride (mg/dL)	103.3 (48.7)
Glucose (mg/dL)	118.3 (27.6)
Hemoglobin A_1_C (%)	7.5 (1.3)
LVEF (%)	55.0 (17.9)

**Table 2:** Outcomes of study patients after EECP treatment

**Characteristics**	**Median (IQR)**		
	**Baseline**	**End of 35**^**th **^**session**	**Follow-up**
	**(n = 30)**	**(n = 27)**	**(n = 30)**
Systolic blood pressure (mm Hg)	122	120	130
	(113–133)	(110–130)	(120–130)
*p-*value		0.139^a^	0.473^b^
Diastolic blood pressure (mm Hg)	75	70	78
	(70–77)	(70–80)	(70–80)
*p-*value		0.839^a^	0.431^b^
Heart rate (beats/min)	70	68	71
	(61–75)	(59–80)	(63–80)
*p-*value		0.628^a^	0.071^b^
Creatinine (mg/dL)	1.02	0.97	1.01
	(0.84-1.36)	(0.8-1.34)	(0.85-1.36)
*p-*value		0.115^a^	0.156^b^
GFR (mL/min per 1.73 m^2^)	70.47	83.46	76.27
	(43.88-89.41)	(47.26-97.51)	(49.02-91.46)
*p-*value		0.075^a^	0.006^b^**
Cystatin C (mg/L)	1.00	0.91	0.94
	(0.78-1.31)	(0.77-1.39)	(0.77-1.27)
*p-*value		0.421^a^	<0.001^b^**
NT-proBNP (pg/mL)	244	200	210
	(120–1067)	(70–653)	(123–398)
*p-*value		0.416^a^	0.425^b^

^a^End of 35^th^ session versus baseline, calculated using Wilcoxon signed ranks test.

^b^Follow-up versus baseline, calculated using Wilcoxon signed ranks test.

(**)indicated statistically significant *p-*value less than 0.01.

Of the study patients, 29 (96.7%) patients had coronary artery disease before EECP treatment followed by hypertension in 27 (90%), diabetes in 23 (76.7%), stroke in 8 (26.7%), CHF in 7 (23.3%) and peripheral artery disease in 4 (13.3%), respectively. Ten (33.3%) patients had smoking history in which 7 (70%) patients were past smokers and 3 (30%) patients were current smokers. The majority (90%, 27/30) of patients had undergone coronary angiography before EECP treatment. Twelve patients (40%) underwent percutaneous coronary intervention and six patients (20%) underwent coronary artery bypass grafting. Of 7 patients with prior history of CHF, 6 had EF < 40% consistent with systolic heart failure due to ischemia and 1 had diastolic dysfunction. According to recent medications before EECP treatment, 93.3% (28/30) of patients received statin therapy, 90% (27/30) aspirin or clopidogrel, 76.7% (23/30) angiotensin-converting enzyme inhibitors (ACEI) or angiotensin receptor blockers (ARB), 63.3% (19/30) beta-blockers, 56.7% (17/30) nitrate and 33.3% (10/30) calcium channel blockers including diltiazem and verapamil. The doses of ACEI, ARB and beta-blockers were unchanged during EECP treatment and no new medications were added during follow- up.

Baseline laboratory findings showed that the mean (SD) of hemoglobin [12.9 (1.8) g/dL], albumin [4.2 (0.4) g/dL] and lipid profiles including cholesterol [146.0 (27.0) mg/dL], triglyceride [103.3 (48.7) mg/dL], HDL cholesterol [49.5 (12.2) mg/dL], LDL cholesterol [77.8 (19.6) mg/dL] were within normal limits except fasting blood sugar [118.3 (27.6) mg/dL] and hemoglobin A_1_C [7.5 (1.3) %] were higher than normal limits. The mean (SD) left ventricular ejection fraction (LVEF) was 55.0 (17.9) % before EECP treatment. The median (IQR) Cr, GFR, Cys C and NT-proBNP before EECP treatment were 1.02 (0.84-1.36) mg/dL, 70.47 (43.88-89.41) mL/min per 1.73 m^2^, 1.0 (0.78-1.31) mg/L and 244 (120–1067) pg/mL, respectively.

During EECP treatment period, there was no withdrawal of EECP treatment or any serious adverse effect occurred. Only two patients reported the development of skin blebs which resolved after supportive treatment and none of the patients had clinical heart failure. After EECP treatment, systolic blood pressure, diastolic blood pressure and heart rate were not significantly changed. Overall, there was an improvement in cardiac symptoms [NYHA Classification Class I/II/III/IV (n): Baseline 0/15/13/2 versus Follow-up 13/16/1/0 (p < 0.001)]. New York Heart Association Classification Class was improved by at least 1 class in 26 patients (86.7%).

In order to evaluate renal function, Cys C and estimated GFR were used as the primary outcomes. At the end of 35^th^ sessions of EECP treatment, Cys C was not significantly different from the baseline [(0.91, 0.77-1.39) mg/L versus (1.00, 0.78-1.31) mg/L, p = 0.421, n = 27 (3 missing data at the end of EECP because of technical error)], but Cys C declined significantly at the end of the follow-up period [(1.00, 0.78-1.31) mg/L versus (0.94, 0.77-1.27) mg/L, p < 0.001, n = 30]. Similarly, GFR was not significantly different at the end of 35^th^ session of EECP treatment [(70.47, 43.88-89.41) mL/min/1.73 m^2^ versus (83.46, 47.26-97.51) mL/min/1.73 m^2^, p = 0.075, n = 27] but GFR significantly increased at the end of the follow-up period [(70.47, 43.88-89.41) mL/min/1.73 m^2^ versus (76.27, 49.02-91.46) mL/min/1.73 m^2^, p = 0.006, n = 30] (Table 2).

In the present study, the majority of patients had coronary artery disease, hypertension and diabetes before EECP treatment and the estimated GFR was also lower than normal population suggesting that several patients had chronic kidney disease (CKD) at baseline. According to the National Kidney Foundation-Kidney Disease Outcomes Quality Initiative (NKF-K/DOQI) workgroup, stage 3 CKD was defined as the presence of GFR <60 mL/min/1.73 m^2^ for at least 3 months [12]. Among patients with stage 3 CKD, most had negative or trace proteinuria and were presumed to have decreased GFR due to nephrosclerosis or decreased renal perfusion. Only one patient had nephrotic range proteinuria and another patient had moderate proteinuria and were both presumed to have diabetic nephropathy. Healthy individuals typically have NT-proBNP ≤125 pg/mL [13,14]. Thus, the parameters including GFR <60 mL/min/1.73 m^2^ and NT-proBNP >125 pg/mL were used as cut -off points for further analysis.

Subgroup analysis showed that patients with baseline GFR <60 mL/min/1.73 m^2^ had significant decrease in Cys C [1.55 (1.26-1.85) mg/L versus 1.4 (1.14-1.65) mg/L, p = 0.004] and corresponding increase in GFR at follow-up after EECP therapy [43.59 (39.1-53.29) mL/min/1.73 m^2^ versus 47.52 (41.1-55.76) mL/min/1.73 m^2^, p = 0.003].

**Figure 1:**
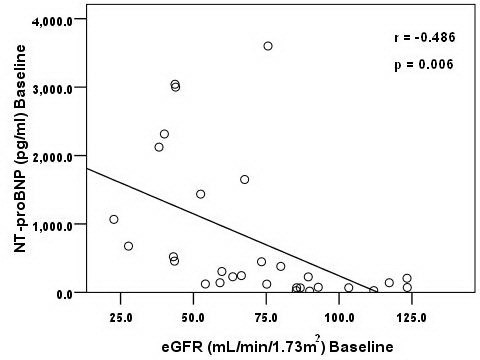
Scatter plot shows relationship between GFR and NT-proBNP at baseline.

There was a significant inverse correlation between GFR and NT-proBNP at baseline (Figure 1). In addition, subgroup with NT-proBNP > 125 pg/mL at baseline had significant decrease in Cys C at follow-up [1.26 (0.94-1.68) mg/L versus 1.14 (0.88-1.51) mg/L, p = 0.001] and correspondingly, had significant increase in GFR [59.43 (43.31-74.47) mL/min/1.73 m^2^ versus 62.26 (46.97-83.18) mL/min/1.73 m^2^, p = 0.001] (Table 3). Of the 30 patients, 10 patients had a decrease in NT-proBNP >30% from baseline at the end of EECP treatment. Among these patients, Cys C was significantly decreased in follow-up period [1.01 (0.81-1.31) mg/L versus 0.97 (0.69-1.20) mg/L, p = 0.017] whereas estimated GFR showed a trend to increase (p = 0.051) (Table 3).

**Table 3:** Change of Cys C and GFR after EECP treatment (n = 30)

**Characteristics**	**Median (IQR)**		
	**Baseline**	**End of 35**^**th **^**session**	**Follow-up**
***Estimated GFR***			
GFR <60 mL/min/1.73 m^2^ (n = 12)	n = 12	n = 9	n = 12
Cystatin C (mg/L)	1.55	1.44	1.4
	(1.26-1.85)	(1.2-1.59)	(1.14-1.65)
*p-*value		0.173 ^a^	0.004 ^b^ **
GFR (mL/min per 1.73 m^2^)	43.59	46.11	47.52
	(39.1-53.29)	(42.06-47.26)	(41.1-55.76)
*p-*value		0.374 ^a^	0.003 ^b^ **
GFR ≥60 mL/min/1.73 m^2^ (n = 18)	n = 18	n = 18	n = 18
Cystatin C (mg/L)	0.83	0.82	0.8
	(0.73-0.89)	(0.72-0.91)	(0.63-0.92)
*p-*value		0.931 ^a^	0.052 ^b^
GFR (mL/min per 1.73 m^2^)	86.05	92.34	88.81
	(75.16-103.3)	(83.46-105.38)	(81.22-109.79)
*p-*value		0.157 ^a^	0.124 ^b^
***NT-proBNP***			
NT-proBNP ≤125 pg/mL (n = 9)	n = 9	n = 9	n = 9
Cystatin C (mg/L)	0.78	0.82	0.81
	(0.76-0.87)	(0.72-0.87)	(0.77-0.87)
*p-*value		0.906 ^a^	0.553 ^b^
GFR (mL/min per 1.73 m^2^)	86.69	88.75	83.11
	(85.28-92.84)	(83.46-105.23)	(81.42-96.54)
*p-*value		0.374 ^a^	0.767 ^b^
NT-proBNP >125 pg/mL (n = 20)	n = 20	n = 17	n = 20
Cystatin C (mg/L)	1.26	1.16	1.14
	(0.94-1.68)	(0.84-1.53)	(0.88-1.51)
*p-*value		0.523 ^a^	0.001 ^b^**
GFR (mL/min per 1.73 m^2^)	59.43	57.66	62.26
	(43.31-74.47)	(46.11-91.46)	(46.97-83.18)
*p-*value		0.246 ^a^	0.001 ^b^**
***NT-proBNP decline >30% from baseline (n = 10 )***	n = 10	n = 9	n = 10
Cystatin C (mg/L)	1.01	1.00	0.97
	(0.81-1.31)	(0.77-1.2)	(0.69-1.2)
*p-*value		0.594 ^a^	0.017 ^b^*
GFR (mL/min per 1.73 m^2^)	65.53	66.87	66.46
	(43.68-79.98)	(47.26-93.98)	(49.02-91.85)
*p-*value		0.214 ^a^	0.051 ^b^

Values are Median (25^th^-75^th^ percentile)*. p-* value was calculated with the use of Wilcoxon signed ranks test, ^a^ End of 35^th^ session versus baseline; ^b^ Last follow-up versus baseline.

(*) indicated statistically significant *p-*value less than 0.05; (**) indicated statistically significant *p-*value less than 0.01.

## Discussion

EECP has been shown to be an effective therapy for patients with angina and CHF [8,15] who were not candidates for revascularization [16]. The safety of EECP treatment was demonstrated in left ventricular dysfunction patients with minor adverse effects such as skin blebs in the present study [16,17]. The prospective evaluation of enhanced external counterpulsation in congestive heart failure (PEECH) study showed that EECP treatment improved exercise tolerance and quality of life [18]. Similar to previous study of Loh et al. [19], the New York Heart Association Classification Class was improved by at least 1 class in 26 patients (86.7%).

In the present study, the majority of patients had diabetes and hypertension co-existing with advanced heart disease. The baseline GFR (70.47 mL/min/1.73 m^2^) was lower than normal population. In such patients, several mechanisms of chronic heart impairment including systolic dysfunction, increased venous congestion and activation of neurohormonal axis might lead to decrease renal function [1]. Previous study has shown that EECP treatment was able to increase GFR by 24% and renal plasma flow by 21-30% in healthy volunteers due to decreased renal vascular resistance, plasma renin activity and endothelin [11,20]. However, there was no study to assess the benefit of EECP treatment on renal function in cardiac patients. Therefore, we conducted the first study to evaluate the immediate and long term effects of EECP treatment on renal function in patients with chronic stable angina and/or CHF.

We found that EECP treatment did not have immediate effect on renal function but improved long term renal function during 16-month follow-up period. The beneficial effect of EECP was greater for those with diminished renal function (GFR <60 mL/min/1.73 m^2^) or high NT-proBNP (>125 pg/mL) at baseline. Among these patients, Cys C decreased approximately 0.12 mg/L which corresponded to increased Cys C–based GFR of 6 mL/min/1.73 m^2^[10]. This finding was consistent with an increase in estimated GFR after EECP therapy using the combined creatinine and Cys C equation (70.47 to 76.27 mL/min per 1.73 m^2^). Regarding two-way interactions of the heart and the kidney, the small improvement in GFR after EECP in this study could benefit for the cardiac function and vice-versa.

Presently, there is consensus on an age-independent NT-proBNP for evaluation of CHF [13,14] and the percentage change of NT-proBNP after therapy is an important predictor of cardiovascular outcomes [21,22]. In the present study, patients with >30% decline of NT-proBNP at the end of 35^th^ session had significantly improved renal function. This was similar to previous study that demonstrated NT-proBNP reduction >30% after therapy to be the best cut-off value for defining the risk of re-hospitalization and mortality [23].

The beneficial effects of EECP on cardiac function have been shown to involve changes in neurohormonal regulation, improved systolic function, endothelial function, and collateral vessel formation [8,24]. It has been proposed that chronic exposure of coronary and peripheral arterial bed to the augmented blood flow and increased shear forces produced by EECP could lead to increased endothelial cell production of nitric oxide (NO), prostacyclin [25] as well as lead to the development of collateral vessels through the release of angiogenic growth factors [26,27] and increased circulating endothelial progenitor cells [28]. In experimental dogs, EECP increased the density of microvessels in the infarcted regions significantly compared with the control group [29]. The formation of new blood vessels(angiogenesis), enhancement of collateral development from preexisting vessels(arteriogenesis), or an improvement in endovascular function [29] may help explain the delay in benefits observed on renal function and the long lasting effects of EECP [15]. Improvement of the results of myocardial perfusion test on EECP therapy had been shown to persist for 3 years [30]. This sustained improvement in cardiovascular function could indirectly account for the long term effects of EECP on GFR observed in our study. The close relationship between the function of the heart and the kidney is supported by the observation that the GFR was inversely related to NT-proBNP level. As this relationship is bidirectional, the small improvement in GFR after EECP observed in this study could be a result of benefits on cardiac function and/or vice-versa.

The effects on renal function were not apparent using serum Cr or Cr-based GFR equation (*data not shown*). Serum Cr can be affected by many factors such as muscle mass and protein intake. It is possible that improvement in cardiac function after EECP might have led to improved nutritional status and masked the small improvement in renal function when serum Cr is used as a marker. Previous studies have suggested that Cys C is a more accurate and sensitive marker of GFR compared to Cr in diabetic and non-diabetic patients [31] as well as being less influenced by age, gender, weight, dietary protein intake and muscle mass, which obscure changes in Cr-based GFR [32,33]. Moreover, changes in Cys C levels can be used to more accurately predict changes in GFR than changes in Cr [34]. Thus, Cys C was used to evaluate renal function in the study. Although Cys C is less dependent on factors such as age, sex, race and body mass index when compared to creatinine-based GFR estimation, it can be affected by other factors such as body composition (lean mass), thyroid dysfunction, cancer and left ventricular mass [35-38]. It is possible that GFR –independent factors could account for decreases in serum cystatin in some individuals, but these factors were not apparent during the follow-up of our patients and were unlikely to account for the changes in serum cystatin C in the group as a whole. In addition, we evaluated the effects of EECP on renal function using GFR estimating equation which employed Cys C in combination with Cr, age, sex and race [10]. This equation has recently been shown to be more accurate than equations using either marker alone [10,39,40] and has been recommended as the optimal equation by experts for assessing CKD [40].

This study has several limitations. This was an observational, small-sized study without a parallel control group or sham EECP group (defined as suboptimal counterpulsation). Routine measurement of LVEF after treatment was not available. We used endogenous markers of renal function rather than formal clearance studies. This would tend to underestimate any changes in renal function especially if the GFR was relatively well preserved. The effects on renal function by EECP as a group were modest, and the clinical significance of such minor improvement in GFR on cardiovascular or renal outcome is uncertain. Given the within-person individual variability of renal function measurements [41,42] and the small size of the study, it may be possible that the apparent statistically significant differences may be explained by chance. On the other hand, it is also possible that if a more sensitive method to detect GFR was available or if only patients with renal impairment or high NT-proBNP were studied, the larger improvement might be detected.

The results from this study provide support for larger comparative studies between EECP and placebo measuring Cys C level and GFR in well-matched groups to be done in the future in order to determine the benefits of EECP treatment. In particular, the observation that the beneficial effect of EECP was greater for patients with diminished renal function and for those with elevated NT-proBNP very much supports the need for such studies in patients with moderate to severe reduction in GFR or in those with congestive heart failure.

## Conclusions

EECP is a safe, effective and feasible treatment for cardiac patients. This is the first study to demonstrate that EECP did not show the immediate effect on renal function but could augment the renal function as measured by Cys C and estimated GFR in cardiac patients after long term follow up, especially in the groups with decreased GFR or high NT-proBNP.

## Abbreviations

ACEI: Angiotensin-converting enzyme inhibitors; ARB: Angiotensin receptor blockers; CHF: Congestive heart failure; Cr: Plasma creatinine; Cys C: Serum cystatin C; EECP: Enhanced external counterpulsation; GFR: Glomerular filtration rate; LVEF: Left ventricular ejection fraction.

## Competing interests

The authors declare that they have no competing interests.

## Authors’ contributions

PR designed the study and wrote the manuscript; NM and OR recruited the patients; PR supervised the EECP treatment; CK helped in the design of the study and in writing manuscript; AC and DP performed the statistical analyses; SA participated in the collection of data and data analysis; SV and AC measured plasma creatinine, serum cystatin C and NT-proBNP; TS critically revised the manuscript. All authors read and approved the final manuscript.

## Pre-publication history

The pre-publication history for this paper can be accessed here:

http://www.biomedcentral.com/1471-2369/14/193/prepub
